# Effectiveness of a low-intensity static magnetic field in accelerating upper canine retraction: a randomized controlled clinical trial

**DOI:** 10.1186/s12903-024-04212-x

**Published:** 2024-04-06

**Authors:** Nataly N. Alqaisi, Rania A. Haddad, Hani M. Amasha

**Affiliations:** 1 Department of Orthodontics, Faculty of Dentistry, Damascus University, Damascus, Syria; 2 Department of Biomedical Engineering, Faculty of Electrical Engineering, Damascus University, Damascus, Syria

**Keywords:** Low-intensity static magnetic field, Magnets, Accelerating orthodontic, Canine retraction, Molar drift

## Abstract

**Introduction:**

Neodymium-iron-boron magnets have been suggested as a contemporary method for accelerating the process of orthodontic tooth movement (OTM).

A limited number of clinical trials evaluated their effectiveness in accelerating OTM which is desirable for both orthodontists and patients.

The present study aimed to investigate the effectiveness of a low-intensity static magnetic field (SMF) in accelerating upper canine retraction movement.

**Materials and methods:**

Seventeen patients (mean age 20.76 ± 2.9 years) with their orthodontic treatment decision to extract the upper and lower first premolars due to bimaxillary protrusion malocclusion were included in this split-mouth study. Canine retraction was performed using Nickel-titanium (Ni-Ti) closed-coil springs (150 g of force on each side). The experimental side received SMF via an auxiliary wire that carried 4-neodymium iron-born magnets with an air gap of 2 mm between the magnets to produce a magnetic field density of 414 mT in the region corresponding to the lateral ligament of the upper canine. To determine the rate of upper canine retraction and upper molar drift, alginate impressions were taken once a month to create plaster casts, which were analyzed digitally via a three-dimensional method.

**Results:**

The rate of upper canine retraction was significantly greater (*P* < 0.05) on the SMF side than that on the control side during the first and second months, with an overall duration (19.16%) that was greater than that on the control side. The peak acceleration occurred during the second month (38.09%).

No significant differences in upper molar drift were detected between the experimental and control sides (*P* > 0.05).

**Conclusion:**

A low-intensity static magnetic field was effective at accelerating upper canine retraction. The difference between the two sides was statistically significant but may not be clinically significant.

The SMF did not affect upper molar drift during the upper canine retraction phase.

**Trial registration:**

The trial was retrospectively registered at the ISRCTN registry (ISRCTN59092624) (31/05/2022).

## Introduction

The introduction of modern therapeutic methods contributes prominently to theories of contemporary orthodontics [1]. Among these, the concept of accelerated OTM is to achieve normal occlusion with shortening treatment period, and minimal side effects on the teeth and periodontal tissue [2, 3]. Various physical methods, such as low-level laser therapy (LLLT) [2], static magnetic field (SMF) [4], and pulsed electromagnetic field (PEMF) [5, 6], are employed in OTM acceleration. Recent animal studies have shown that the SMF may possibly shorten the duration of orthodontic treatment [4, 7].

Similarly, histological studies have revealed that alveolar bone remodeling process is likely to be activated under the influence of magnetic fields, as the activity of osteoblasts increases new bone deposition on the tension side [1, 8]. Magnetic fields have the advantage of being able to cross both the mucous membrane and bone [9]; notably, they do not require patient cooperation [10].

The recent development of small magnets made of new, powerful permanent magnetic alloys (neodymium- iron- boron) also known as rare earth magnets, has led to an increase in the use of magnets [11]. Such magnets are 20 times stronger than the magnets used previously; thus, they can be used at a size 20 times smaller than the previous ones with the same amount of resulting force. This feature made them suitable for the use inside the oral cavity easier and more comfortable for patients [11]. The magnetic flux density was measured with a Tesla (T) or Milli-Tesla (mT) [11].

However, few studies in the literature have examined the effect of static magnetic field (SMF) therapy on the rate of OTM in short-term animals [4, 7]. The SMF has been utilized in various randomized controlled clinical trials (RCTs) for orthodontic treatment, such as correction of Class II malocclusion [12], correction of Class III malocclusion [13], and closure of midline diastemas [14].

On the other hand, recent studies have suggested that a static magnetic field can enhance the effectiveness of anticancer drugs while minimizing cytotoxicity and side effects [15–17].

Accordingly, the primary objective of this study was to investigate the effectiveness of a low-intensity static magnetic field (SMF) in accelerating upper canine retraction movement and reducing treatment duration. The secondary aim was to evaluate upper molar drift following canine retraction.

Null hypothesis (H0): Applying a static magnetic field does not lead to the acceleration of upper canine retraction or upper molar drift in the context of orthodontic treatment.

## Materials and methods

### Ethics approval and patient consent

This prospective, randomized, single-center, single-blinded, split-mouth study was approved by the institutional review board and the ethical review committee of Damascus University (no. 906/22-11-2021) and was retrospectively registered at the ISRCTN registry (identifier: ISRCTN59092624). This randomized clinical trial with a split-mouth design was written according to the Consolidated Standards of Reporting Trials (CONSORT) statement [18]. Patient recruitment started in August 2021 and ended in November 2021.

Each maxilla was randomly split into two halves (the experimental side, on which a static magnetic field (SMF) was applied; and the contralateral side, not receiving the SMF and serving as a control).

### Sample size calculation and participants

G*power 3.1.9.7 software (Universität Düsseldorf, Düsseldorf, Germany) was used to calculate the sample size based on the speed of upper canine retraction using the mean and standard deviation from a prior related study [19], with the following assumptions; a paired t-test with a power of 95%, a significance level of 0.05, and an effect size of 0.93. Consequently, the calculated sample size was 17 patients (34 canines).

### Participants and eligibility criteria

The treatments were performed by the same orthodontist (N.A). Clinical examination, intraoral and extraoral photographs, dental casts, and radiographic records were taken before starting orthodontic treatment for 30 patients referred to the Department of Orthodontics. Eighteen patients met the inclusion criteria. Only one of them has withdrew for travel reasons. All patients fulfilled the following inclusion criteria: Adult patients (18–28 years old) with full permanent dentition, bimaxillary protrusion, dental class I relationship of the canines and molars according to Angle classification, class I or II skeletally, normal or excessive facial height (Bjork > 390֯), healthy periodontal tissues and reasonable oral health (Plaque index ≤ 1), no previous orthodontic treatment, and nonsmokers. The subject exclusion criteria: were patients suffering from systemic diseases or syndromes, and those who had impacted teeth.

Informed consent was obtained from all patients who met these criteria.

#### Randomization, allocation concealment, and blinding

Simple randomization was conducted by using random computer-generated numbers (Minitab Statistical Software: version 20) with an allocation ratio of 1:1 performed by one of the academic staff (not involved in this research) at the Department of Orthodontics.

The allocation sequence was concealed using sequentially numbered, opaque, closed envelopes, which were opened only before the beginning of upper canine retraction.

Patient and practitioner blinding was not applicable. Therefore, blinding was applied only for the outcome assessor while recording plaster-distributed casts with serial numbers to ensure blinding and avoid bias in the investigation, after removing the magnetic appliance for taking the impressions at all times of investigation.

### Intervention

**Fig. 1:**
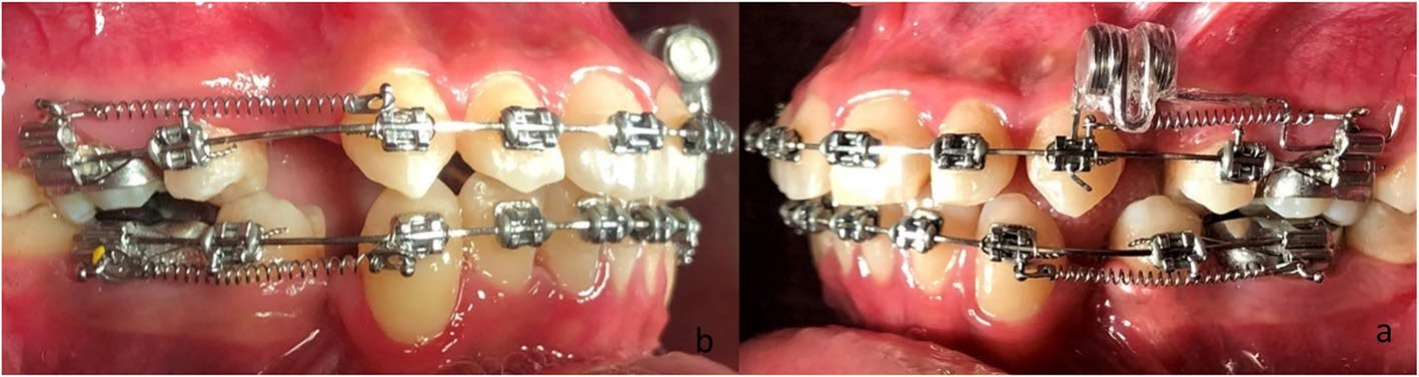
**a** The experimental side in which the static magnetic field was applied on the left side of the maxilla. **b** The contralateral side served as a control

In the initial phase, the soldered transpalatal arches were placed as moderate anchors, before extracting the upper and lower first premolars. One week later, leveling and alignment were started using a straight wire, ‘0.022’ slot MBT appliance (Mini Master Series, American Orthodontics, Sheboygan, USA) with the following arch wire sequence: 0,014-in. NiTi, 0.016-in. NiTi, 0,016 × 0,022-in. NiTi, 0,017 × 0,025-in. NiTi, 0,019 × 0,025-in. Stainless steel (S.S.) (the basal arch-wire) was used. The second phase was one month after applying the basal wire when an alginate impression of the maxillary arch was taken. Then upper and lower canine distalization was started using NiTi closed-coil springs (Jiscop, Gunpo-si, Korea) extended from the hook of the molar band to the hook of the canine bracket. The generated force was calibrated using an intraoral dynamometer (040-711-00, Dentaurum, Ispringen, Germany). The medium force of the Ni-Ti closed coil spring was calibrated and readjusted when necessary to maintain it at a 150 g level during the whole retraction phase. The spring was replaced with a shorter spring when needed. For the experimental side, the acceleration of upper canine retraction was investigated by applying a static magnetic field through an auxiliary wire carrying the magnet (Fig. 1).

### Application of a static magnetic field

**Fig. 2:**
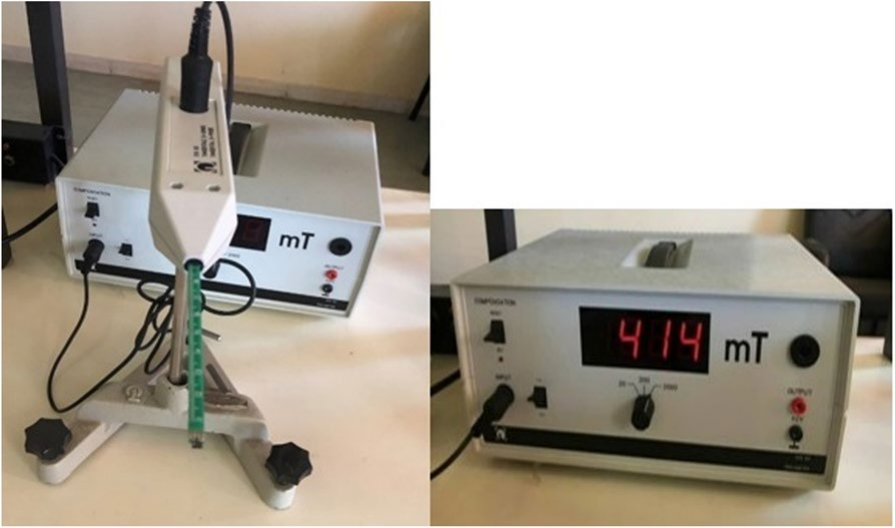
A Digital Tesla meter with a Hall probe device was used

**Fig. 3:**
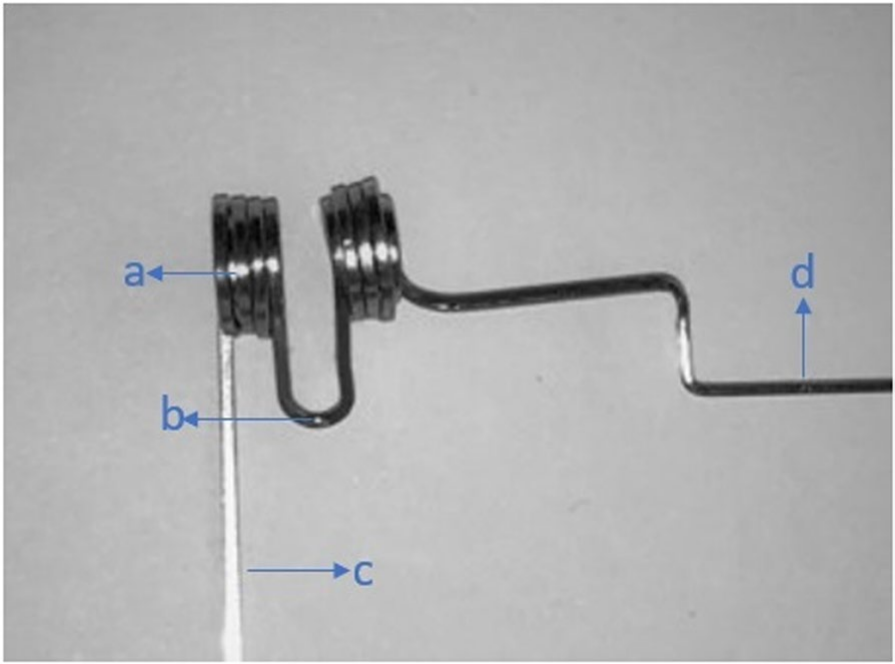
The auxiliary wire carrying the magnet: **a** Two magnets are placed on each side of the circular rings. **b** The loop between the magnets is 2 mm long to represent the air gap between the magnets. **c **The auxiliary wire carrying the magnet is fixed by the vertical slot of the upper canine bracket. **d** The auxiliary wire carrying the magnet was inserted into the auxiliary tube of the upper molar band

**Fig. 4:**
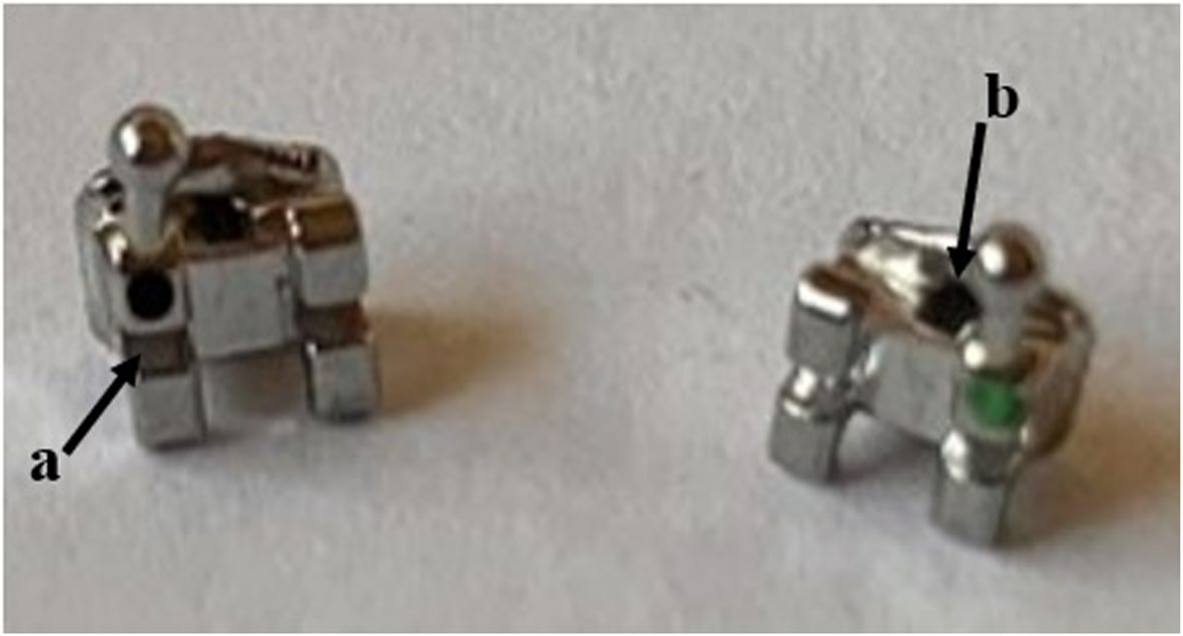
MBT 0.022 Upper canine bracket: **a** The horizontal slot. **b** The vertical slot

The magnetic appliance used for accelerating upper canine retraction was as follows:

- A- Four Neodymium-iron-boron (Nd-Fe-B) magnets without cobalt, which can maintain magnetism permanently and are coated with titanium and circularly shaped materials with a diameter of 4 mm and a height of 1 mm, were used (Magnet Expert).
- B- Straight S.S. wire 0.016 × 0.022-in.
- C- MBT 0.022-in upper canine brackets with a vertical slot.
- D- Circular rings, carrying the magnet, formed on an auxiliary 0.016 × 0.022-in. S.S straight wire, by a Tweed plier so that the 4 mm diameter neodymium magnets could be placed inside the matched circular rings (two magnets at each pole). The auxiliary wire contained a loop with a distance of 2 mm between the poles to represent the air gap between the magnets so that they produced a magnetic field density of 414 mT in the region corresponding to the lateral ligament of the upper canine. The magnetic field density was measured by a Digital Tesla meter (Dexing Magnet Tech. Co., Ltd.) with the Hall probe device which is an electronic device that allows accurate measurement of the intensity of the magnetic field with a 1 mm diameter probe placed between the magnets (Fig. 2). The auxiliary wire carrying the magnet was fixed between the vertical slot of the upper canine bracket and the auxiliary tube of the upper molar band (Fig. 3), with the appliance moves freely within the auxiliary tube of the molar band and applies no forces; thus, there are no differential friction implications between the experimental and control sides because the vertical slot is not connected with the horizontal slot of the base wire (Fig. 4). The auxiliary wire carrying the magnet was covered with flexible vacuum-formed sheets of 0.5 mm thickness.

### Follow-up during canine retraction

Patients were followed up through the canine retraction phase once a month, during which the spring was reactivated to maintain a force of 150 g on both sides.

The springs and auxiliary wire carrying the magnet were monitored periodically every 2 weeks, and alginate impressions were taken once a month.

The total follow-up period started with the onset of canine retraction and was concluded when canine retraction was accomplished.

### Outcome measures

The rate of canine movement was the primary outcome measurements, and the upper molar drift was the secondary outcome measurements.

The rates of upper canine retraction and upper molar drift were recorded once a month using plaster casts and analyzed in a three-dimensional manner. Transferred scanned casts were analyzed digitally (Exocad - Dental CAD 3.1 Rijeka). A digital scanner (MEDIT, T710, Seoul, South Korea), which has a high accuracy of 11 microns, was used. The accuracy and reliability of this method have been proven by previous studies [20–22].

Dental casts were obtained at five time points: (T0) before canine retraction, (T1) after one month, (T2) after two months, (T3) after three months, and (T4) after four months. A T-Final was considered for each patient when one of the upper canines was fully retracted.

**Fig. 5:**
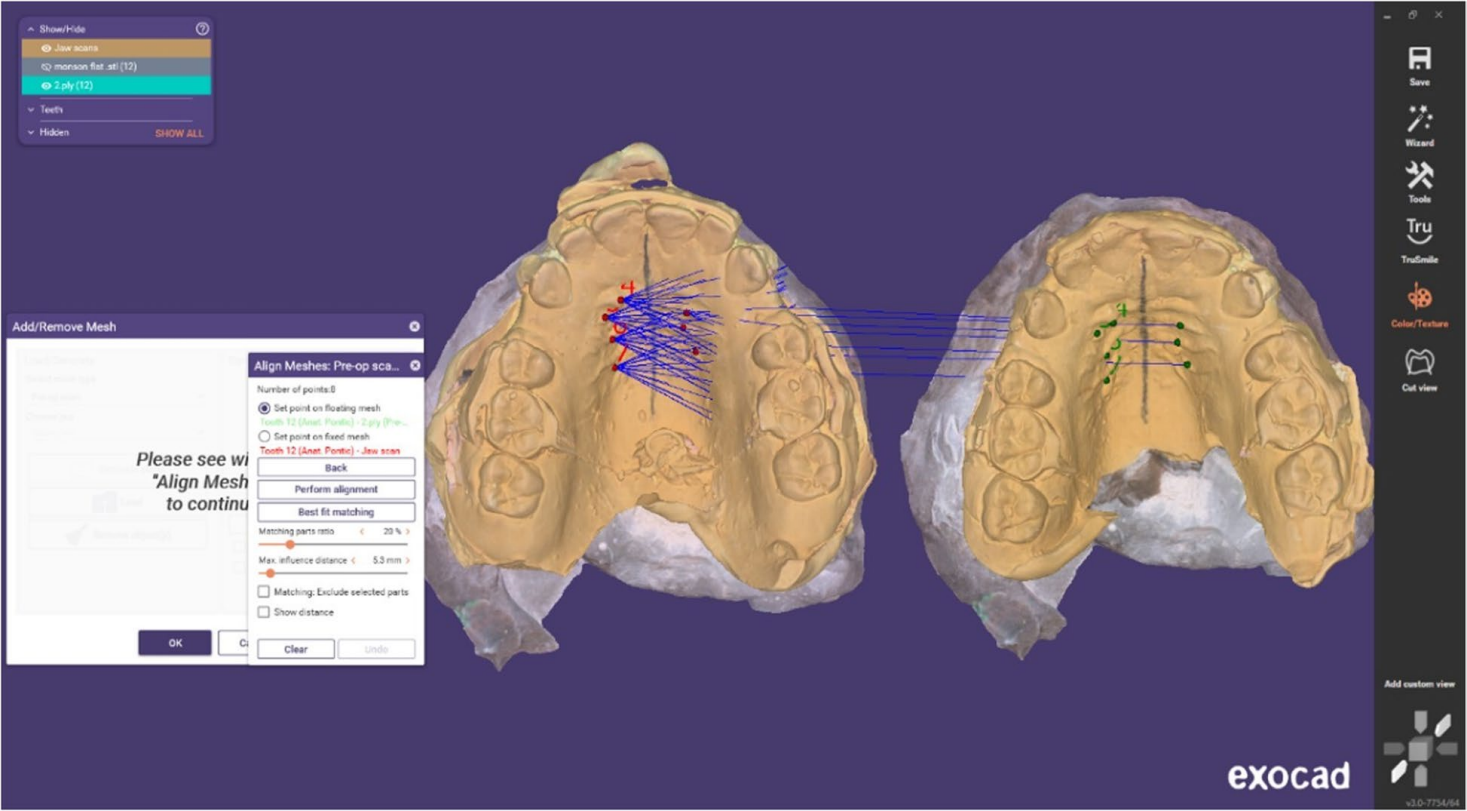
Superimposition was performed between each cast and the next cast depending on the palatal rugae region

**Fig. 6:**
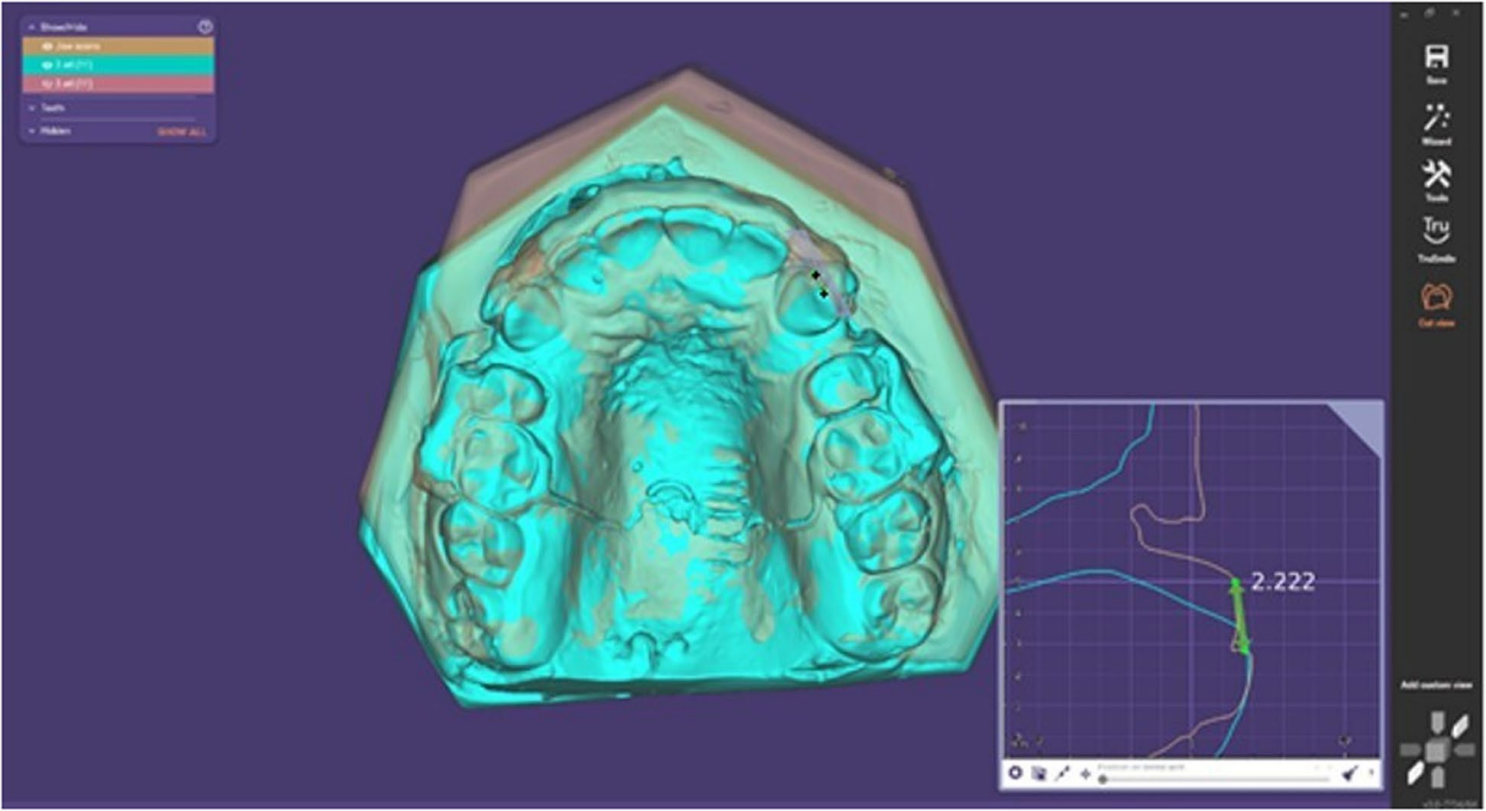
The distance between the two cusps was measured occlusally to evaluate anterior-posterior canine movement

Superimposition was performed between each cast and the next cast at the measuring time points (T0-T1, T1-T2, T2-T3, T3-T4, and T0-Tf), depending on the palatal rugae region as a fixed area [23] (Fig. 5). The cusp tip of the upper canine was determined, and the distance between the two cusps was measured occlusally to evaluate anterior-posterior canine movement (Fig. 6); then, the speed of upper canine retraction was calculated (mm/month).

To evaluate molar anchorage loss, superimposition was performed between each cast and the next cast at specific time points [24] (T0-T1, T1-T2, T2-T3, T3-T4, and T0-Tf), using the palatal rugae region as a fixed area [23].

**Fig. 7:**
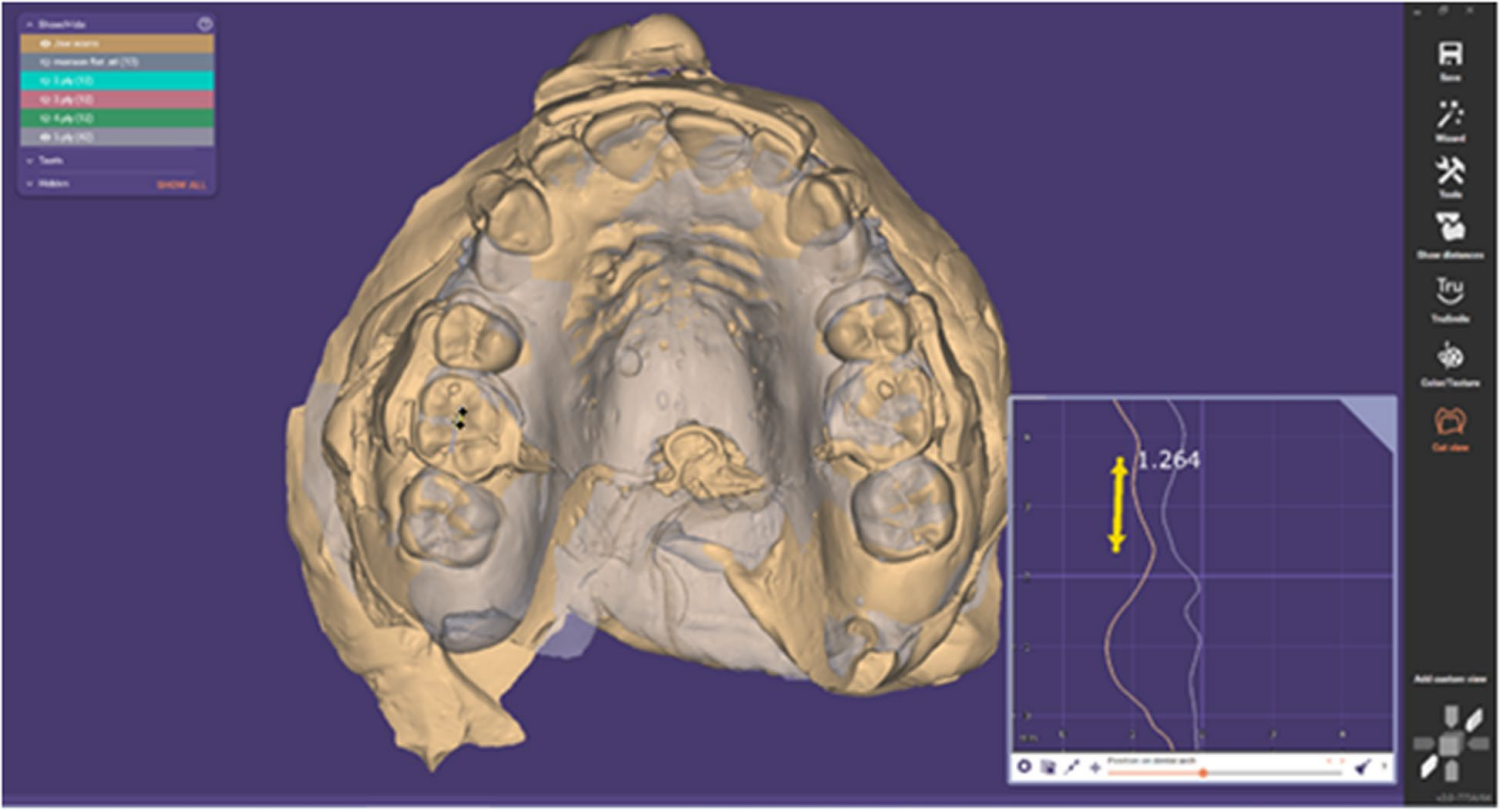
The distance between the two central fossa was measured occlusally to evaluate anterior-posterior movement of the maxillary first permanent molars

The central fossa of the maxillary first permanent molars was identified at each time point, and the distance between the two central fossae was measured occlusally to determine the amount of anterior-posterior movement of the maxillary first permanent molars (Fig. 7). Subsequently, the speed of upper molar drift was calculated (mm/month).

### The error of method

One month after the first measurement, twenty casts were randomly chosen to calculate the method error for the recorded measurements. The supper imposition was repeated, all reference points were redetermined, and the entire measurements were recorded using the same program (Exocad - Dental CAD 3.1 Rijeka).

A paired t test was conducted to evaluate systematic errors (investigating validity). Moreover, there was no significant difference (*P* < 0.05) between the two measurements (Table 1).

**Table 1:** Assessment of systematic error in the current study (*n* = 20 casts) using paired t test

	(SD) 1st measurement	(SD) 2nd measurement	Mean Difference	The confidence interval 95%		*t* value	*P* value
				Lower	Upper		
C.Disp	1.70	1.70	0.00	0.00357-	0.00697	0.676	0.507 (NS)

*SD* Stander deviation, *C.Disp *Canine displacement, *NS* No significant difference

The intraclass correlation coefficient (ICC) was also calculated to evaluate random errors (to investigate reliability). The results of this test showed that ICC > 0.999 (Table 2).

**Table 2:** Interclass correlation coefficients of repeated measurements in the current study (*n* = 20 casts)

	ICCs	The confidence interval 95%		*f* value	*P* value
		Lower	Upper		
C.Disp	1.000	0.999	1.000	7131.551	0.000***

*C.Disp* Canine displacement

*** Significant at *P* < 0.001

### Statistical analysis

The statistical analysis was performed using SPSS for Windows (version 26.0; SPSS, Chicago, USA).

All the data were analyzed on both sides with the Shapiro Wilk test to assess their distribution.

The data for the upper canine retraction variables were subjected to a normal distribution, and a paired sample t test was used.

The data for the upper molar drift variables were subjected to a nonnormal distribution, and a Wilcoxon signed-rank matched pairs test was used.

## Results

**Fig. 8:**
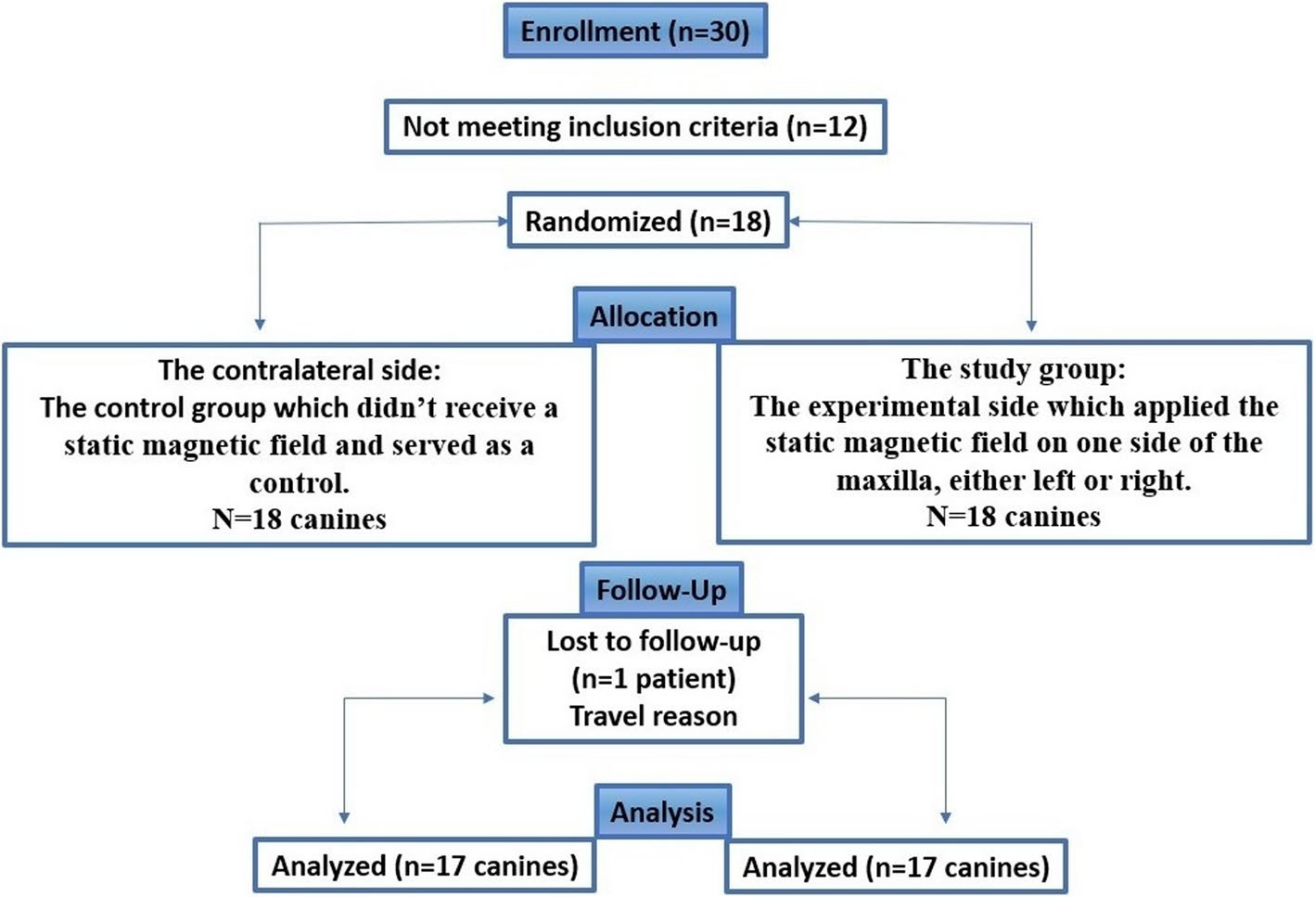
CONSORT Participant flow diagram

The final study sample consisted of 17 patients (13 females, 4 males), with an average age of 20.76 ± 2.9 years; these patients were distributed among the same patients on the experimental and control sides. The CONSORT flow diagram of patient recruitment, follow-up, and entry into the data analysis is given in (Fig. 8).

Patient recruitment started in August 2021 and ended in November 2021.

**Fig. 9:**
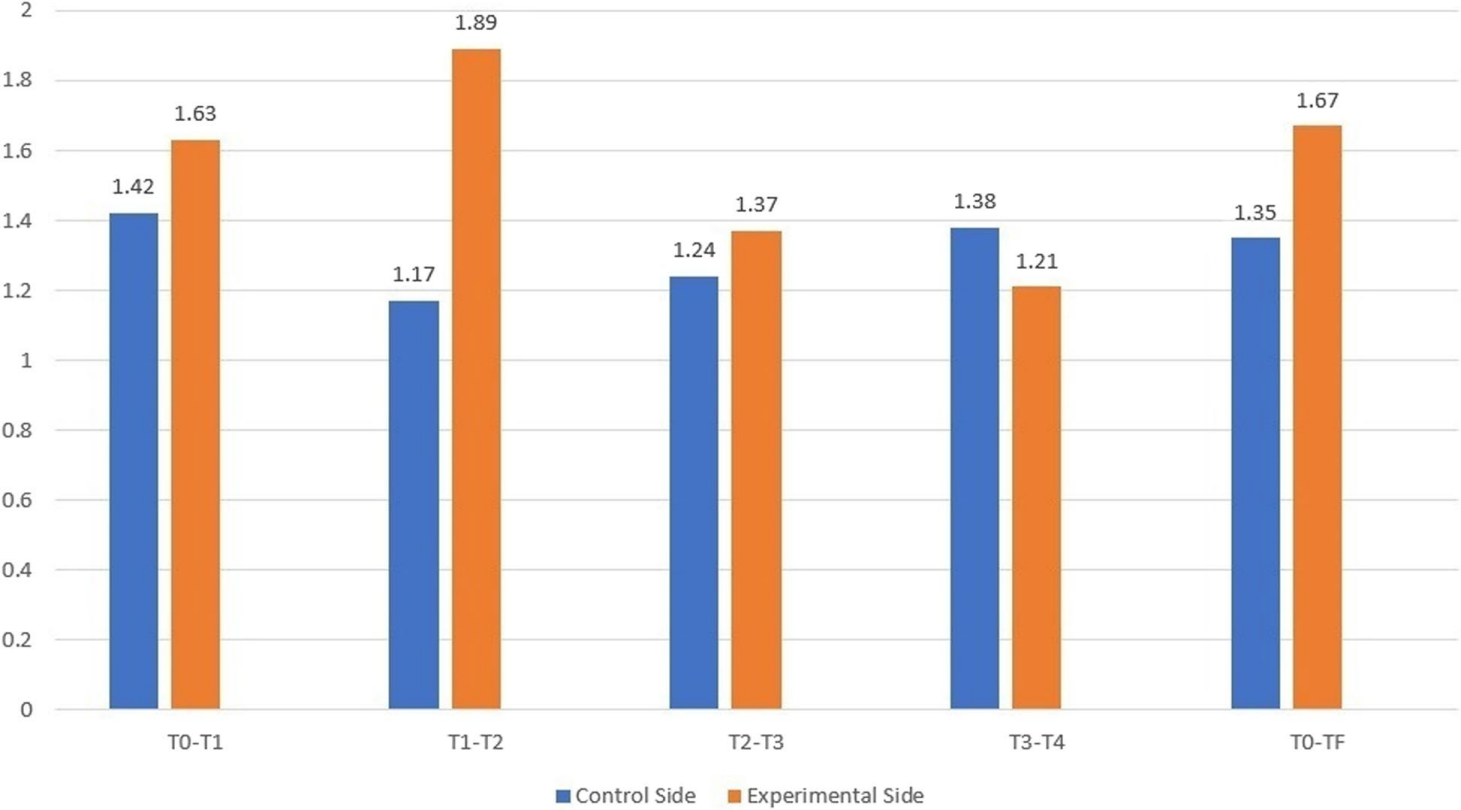
Differences in the mean speeds of upper canine retraction on both the control and experimental sides

Table 3 results of statistical tests for the difference in upper canine retraction. A significant difference was detected between the control and experimental sides during the first month (0.019 < 0.05), the second month (0.000 < 0.05), and overall duration (0.000 < 0.05). However, the acceleration rates were 12.88%, 38.09%, and 19.16%, respectively. The peak of speed acceleration occurred during the second month (38.09%). At the third and fourth months, there was no significant difference between the control and experimental sides (*P* > 0.05). The mean differences in upper canine retraction speed between the control and experimental sides are expressed monthly in (Fig. 9).

**Table 3:** The results of statistical tests for the difference in upper canine retraction between both sides (mm/month)

Time	**Variable**	**n**	Mean	**Std. deviation**	Mean difference	Stander deviation difference	**The confidence interval 95%**		*t* value	*P* value
							Lower	Upper		
T0-T1	**C.RetS.C**	17	1.42	0.661	-0.208	0.33	-0.38	-0.04	-2.601	0.019*
	**C.RetS.E**	17	1.63	0.592						
T1-T2	**C.RetS.C**	17	1.17	0.606	-0.721	0.59	-1.03	-0.42	-5.001	0.000***
	**C.RetS.E**	17	1.89	0.507						
T2-T3	**C.RetS.C**	14	1.24	0.613	-0.124	0.42	-0.36	0.12	-1.117	0.2840 **(NS)**
	**C.RetS.E**	14	1.37	0.493						
T3-T4	**C.RetS.C**	4	1.38	0.35	0.1640	0.65	-0.87	1.19	0.505	0.6490 **(NS)**
	**C.RetS.E**	4	1.21	0.489						
T0-Tf	**C.RetS.C**	17	1.35	0.41	-0.992	0.16	-0.40	-0.24	-6.64	0.000***
	**C.RetS.E**	17	1.67	0.43						

T0-T1: 1st month after upper canine retraction, T1-T2: 2nd month after upper canine retraction, T2-T3: 3rd month after upper canine retraction, T3-T4: 4th month after upper canine retraction, T0-TF: the overall duration

C.RetS.C: Canine retraction speed control side (mm/month)

C.RetS.E: Canine retraction speed experimental side (mm/month)

*NS* No significant difference

* Significant difference at *P* < 0.05, *** Significant difference at *P* < 0.01. Using a paired t test

The results of statistical tests revealed the difference in the speed of upper molar drift between both sides is shown in (Table 4). Moreover, there was no significant difference between the control and experimental sides regarding upper molar drift (*P* > 0.05) at the 95% confidence level, throughout the overall duration.

**Table 4:** The results of statistical tests for the difference in upper molar drift between both sides (mm/month)

**Time**	**Variable**	**n**	**Minimum**	**Maximum**	**Mean**	**Std. deviation**	**z**	*P* value
T0-T1	M.DriftS.C	17	0.0	0.96	0.35	0.33	− .594	0.552 (NS)
	M.DriftS.E	17	-0.2	0.89	0.28	0.33		
T1-T2	M.DriftS.C	17	0.0	1.57	0.52	0.59	− .663	0.508 (NS)
	M.DriftS.E	17	-0.3	1.89	0.43	0.64		
T2-T3	M.DriftS.C	14	0.0	0.97	0.41	0.35	− .445	0.657 (NS)
	M.DriftS.E	14	0.0	1.62	0.35	0.49		
T3-T4	M.DriftS.C	4	0.0	0.73	0.23	0.34	-1.342	0.180 (NS)
	M.DriftS.E	4	0.0	0.18	0.07	0.09		
T0-Tf	M.DriftS.C	17	0.0	1.01	0.41	0.30	− .982	0.326 (NS)
	M.DriftS.E	17	0.0	0.81	0.32	0.23		

T0-T1: 1st month after upper canine retraction, T1-T2: 2nd month after upper canine retraction, T2-T3: 3rd month after upper canine retraction, T3-T4: 4th month after upper canine retraction, T0-TF: the overall duration

M.DriftS.C: Molar drift speed control side (mm/month)

M.DriftS.E: Molar drift speed experimental side (mm/month)

*NS* No significant difference, using a Wilcoxon signed-rank matched pairs test was used

### Harms

Patients were asked to report any side effects (such as pain, discomfort, swelling, etc.) they suffered at each appointment. None of our patients reported any such complaints.

## Discussion

To the best of our knowledge, this is the first trial in the literature evaluating the effectiveness of low-intensity therapy (SMF) in accelerating retraction of the upper canine and in upper molar drift. Thus, the comparison of the current results with those in the literature is limited. The design of the current study follows the most common ones in clinical research which is the randomized controlled clinical trial of split-mouth design, that greatly reduces the impact of individual interventions on treatment efficacy [25].

Premolar extraction was conducted at the beginning of treatment and before appliance fitting to avoid the extraction effect of the regional acceleration phenomenon (RAP) [26].

NiTi closed coil springs were used to retract canines because of the continuous light force they generate and providing better oral health than elastomeric chains [27].

The accelerating magnet device used was placed buccally so that the magnetic field was in the area corresponding to the lateral ligament of the upper canines, because of its proven effectiveness in activities of cells in periodontal tissues by causing faster absorption and deposition of bone [7, 28]. The appliance was comfortable as none of the patients reported any discomfort, besides it did not obstruct the movement of the tongue or speech (which were investigated as a part of the study).

The auxiliary wire carrying the magnet was covered with flexible vacuum-formed sheets 0.5 mm in thickness to secure a smooth surface with soft tissues for the patient’s ultimate comfort and to protect the magnets from corrosion and saliva.

The speed of upper canine retraction and anterior-posterior molar movement, on both the control and experimental sides were evaluated using a digital scanner to perform measurements via a computer program (Exocad - Dental CAD 3.1 Rijeka). The accuracy and reliability of this method were proven previously in the literature [20, 21].

The results of this study showed that the speed of upper canine retraction on the experimental side was significantly greater than that on the control side during the first and second months and during the overall duration of retraction (*P* value < 0.05). The null hypothesis (H0), which stated that the SMF does not accelerate upper canine retraction, was rejected. These findings are in agreement with the findings of Daskalogiannakis and McLachlan [19], who reported similarly that the SMF is effective at accelerating upper canine retraction. However, there the difference in the rate of acceleration might be due to the different magnets used, which affects the intensity of the magnetic field.

Additionally, our results in the timing of the peak acceleration that occurred during the second month agreed with those of Darendeliler and Sinclair [7] and Sakata et al. [4]. Likewise, their experimental studies on animals showed that the application of SMF can accelerate OTM during the early period.

Similarly, Bhad Patil and Karemore [5] confirmed that PEMF therapy is effective at accelerating upper canine retraction despite differences in the magnetic field applied.

However, the results of the present study did not agree with the findings of Tengku et al. [29], who used different magnetic field intensities (100–170 Gauss) on rats and found that a static magnetic field does not enhance OTM.

The results of this study revealed that the upper molar drift on the experimental side was lower than that on the control side during the overall duration of treatment. However, no significant difference was observed (*P* > 0.05) between both sides.

These findings suggest that the SMF did not affect upper molar drift during the upper canine retraction phase. Therefore, we accept the null hypothesis (H0). After reviewing the literature, we did not find any study that investigated the speed of upper molar drift accompanying upper canine retraction with the application of a SMF to compare the results of the current study with it.

### Limitation

Patient and practitioner blinding was not applicable. Therefore, blinding was applied only for the outcome assessor when recording the casts. This might be considered a limitation of this study, but this was not possible due to the clarity of the magnets; moreover, to ensure the comfort of patients, the placebo device was not applied on the control side.

## Conclusion

A low-intensity static magnetic field was effective at accelerating retraction of the upper canines during the first and second months and for the overall duration of the retraction. The difference between the two sides was statistically significant but may not be clinically significant.

The SMF did not affect upper molar drift during the upper canine retraction phase.

### Trial registration

The trial was retrospectively registered at the ISRCTN registry (ISRCTN59092624) (31/05/2022). (10.1186/ISRCTN59092624).

## Data Availability

The data used and analyzed during the current research are available from the corresponding author upon request.
